# Development of Potential Pharmacological Targets to Normalize Gene Expression in Islets of Type 2 Diabetic Patients

**DOI:** 10.2174/0109298673352470250312082922

**Published:** 2025-04-07

**Authors:** Viridiana Basaldúa-Maciel, Fernando Martínez-Esquivias, Juan Manuel Guzmán-Flores

**Affiliations:** 1 Doctorado en Biociencias, Centro Universitario de Los Altos, Universidad de Guadalajara, Tepatitlán de Morelos, Jalisco, México;; 2 Departamento de Ciencias Pecuarias y Agricolas, Centro Universitario de Los Alto, Universidad de Guadalajara, Tepatitlán de Morelos, Jalisco, México;; 3 División de Ciencias Biomédicas, Departamento de Ciencias de la Salud, Centro Universitario de los Altos, Universidad de Guadalajara, Tepatitlán de Morelos, México

**Keywords:** Type 2 diabetes, pancreas, experimental drugs, multi-omics, bioinformatics, pharmacology

## Abstract

**Background:**

Type 2 diabetes (T2D) is a disease of high prevalence that is expected to continue increasing despite the pharmacological treatments available; in most cases, it is difficult to control. Therefore, more research on experimental drugs is necessary to propose better treatments.

**Objective:**

This study aimed to identify the molecular alterations of pancreatic islets in type 2 diabetes through multi-omics data integration and possible pharmacological targets using bioinformatics methods.

**Methods:**

In this study, the OmicsNet tool was used to integrate the multi-omics data associated with T2D, and the protein-protein interaction was visualized. Then, gene ontology and KEGG pathways analyses were carried out. Using the DrugRep server, the hub genes obtained underwent a virtual screening with experimental drugs, and twelve experimental drugs were selected to execute the molecular docking by CB-Dock2. Finally, the interactions were displayed in BIOVIA software.

**Results:**

Our results showed that the main molecular alterations of pancreatic islets in T2D were enzyme binding, mitochondrial metabolism, transcription factors, *etc.* They were involved in glucose uptake, receptor insulin signaling, and secretion. The molecular docking showed that SRC, AKT1, CREBBP, and HSP90AA1 were therapeutic targets for DB02729, DB04877, DB07970, DB07789, and DB03373.

**Conclusion:**

We identified some alterations in the pancreas of patients with T2D, ten hub genes, and five experimental drugs that could potentially correct gene expression abnormalities. However, further studies are required to validate these results.

## 1 INTRODUCTION

Type 2 diabetes (T2D) is a heterogeneous disease, in which various genetic and environmental factors contribute to progressive loss of β-cell mass and function that manifests clinically as hyperglycemia with the risk of developing chronic complications [1]. The pathophysiology of T2D is primarily driven by the induction of skeletal muscle, hepatic, and adipose insulin resistance attributed to ectopic lipid accumulation due to obesity, a process in which the immune system is also involved [2, 3]. Obesity and reduced physical activity are states of insulin resistance, and together with genetic factors, they impose considerable strain on pancreatic beta cells, requiring them to increase insulin secretion to compensate for the impaired insulin action [4]. Over recent decades, chronic low-grade metabolic inflammation, both local and systemic, referred to as metainflammation, has been recognized as a contributing factor to the onset of insulin resistance and secretion, leading to the progression of T2D [5, 6]. Extensive research has provided valuable insights into how inflammatory pathways regulate glucose homeostasis, where the composition of the gut microbiota, the immune system, and glucometabolic pathways play a pivotal role [7, 8]. The combined elevation of IL-6 and IL-1β and elevated levels of TNF-α increase the risk of developing T2D [9], and the overexpression of MCP1 in beta cells promotes macrophage infiltration, promoting insulin resistance and beta cell dysfunction [10]. Furthermore, genetic variations in anti-inflammatory cytokines like IL-4 have been associated with T2D susceptibility [11].

The incidence of T2D is expected to continue increasing worldwide. Mexico is the fourth country with the highest prevalence of type 2 diabetes. In 2022, 18.3% of the 82 million individuals aged 20 and older were living with T2D, with 31.2% of them undiagnosed. Furthermore, 13% of all deaths were attributed to this condition, and the prevalence of the disease is projected to rise by 60% by 2050 [12].

Plasma insulin response to the development of insulin resistance typically increases during the natural history of T2D, which does not mean that the beta-cell functions normally [3]. Studies in cell lines and mouse models of diabetes have revealed an increase in beta- cell oxidative stress, endoplasmic reticulum (ER) stress, and amyloid deposition that will lead to beta- cell exhaustion due to prolonged elevations in glucose metabolism and insulin production, ultimately causing beta-cell death through apoptosis due to glucose/lipotoxicity [13, 14]. This loss cannot be endogenously compensated with newly generated beta-cells owing to the incapability of neogenesis and replication of the human pancreas after age thirty [15].

Multi-omics integrative approaches have been critical in predicting disease risk, disease subtyping and classification, biomarker discovery, deriving biological insights, and stratifying patients for therapy [16]. Recently, with the development of omics technologies and bioinformatics, multi-omics research on glycolipid metabolism disorder has increased, which is conducive to understanding the molecular mechanisms of disease occurrence and evaluating biomarkers [17, 18]. Therefore, it has been found that the genes TCF7L2, PPARG, KCNJ11, SLC30A8, and FTO regulate the susceptibility to glycolipid metabolism disorders [19] and some other essential findings in non-coding RNAs, proteomics, metabolic markers, and gut microbiota.

T2D management requires lifestyle behavior changes and pharmacological treatments to delay complications and maintain a quality of life [20]. Still, despite these benefits due to the increasing number of diabetes cases worldwide, there is an international concern to provide even more effective treatments to control this condition [21, 22], where the discovery of new antidiabetic drugs is still an essential field of study in medicine. Several studies have demonstrated that computational tools, such as virtual screening and 3D-QSAR, can aid the development of new drugs with reduced adverse side effects [23]. Molecular docking is one of the most used methods for rapid screening and finding the effective composition [24] consisting of a simulation approach that predicts the molecules that bind properly to an enzyme that could reduce the cost of research by diminishing the random trial and error and animal sacrifice [25].

Therefore, this study aimed to identify the molecular alterations of pancreatic islets in T2D through a multi-omics data approach, such as genes, mRNA, and proteins, and obtain the hub genes to propose possible pharmacological targets by molecular docking.

## 2 MATERIALS AND METHODS

### 2.1 Genes Associated with Type 2 Diabetes

Type 2 diabetes-associated genes were extracted from three publicly available databases: DisGeNET [26], Malacards [27], and Comparative Toxicogenomics Database (CTD) [28]. Using the “search” tool and the “disease” option, the term “type 2 diabetes” was searched in DisGeNET, while in Malacards, through the “explore a malady” tool, the term “type 2 diabetes mellitus” was searched, and the “genes” option was selected. For the CTD database, the options “search,” “genes,” “gene-disease association,” and “all” were chosen under the term “diabetes mellitus, type 2”. From these searches, 3260 genes related to T2D were obtained. All searches were limited to the species *Homo sapiens*.

### 2.2 mRNA Associated with Type 2 Diabetes

The mRNA data were obtained from the GEO (Gene Expression Omnibus) platform [29]. We performed a search with the keyword “diabetes” and then filtered the results for the species *Homo sapiens*. We then reviewed the results and selected only those who took pancreas samples from subjects with T2D. Finally, we obtained three datasets. The design of GSE20966 involved analyzing the expression profiles of beta cells from cadaveric pancreases of 10 control and 10 T2D subjects. Laser capture microdissection was used to isolate the cells, and the data were normalized and analyzed using DNA-Chip analyzer software. GSE25724 dataset of human islets was isolated from the pancreas of six T2D subjects and seven controls by collagenase digestion, and microarray analysis was performed. Then, GSE38642 islets were obtained from cadaver donors, 54 controls, and 9 T2D subjects by the Nordic Islet Transplantation Programme, and the microarrays were performed using GeneChip Human Gene 1.0 S whole transcript according to Affymetrix standard protocol. Before these datasets were analyzed with the GEO2R tool to explore DEGs between T2D and normal pancreatic beta cell samples, a total of 6918 mRNA was retrieved after unifying the results of the three datasets and eliminating duplicates. Statistically significant DEGs were defined with logFC ≥2; the *p*-value < 0.05 was the cut-off criterion.

### 2.3 Proteins Associated with Type 2 Diabetes

The Omics Discovery Index (OmicsDI) [30] is a platform for searching and linking omics public data. The selection criteria were proteomic data and pancreatic tissue of T2D disease. We obtained five results, of which only one was selected [31], where samples of pancreatic islets were retrieved from 43 multi-organ donors. The World Health Organization guidelines were followed to identify T2D subjects based on the percentage of glycosylated hemoglobin in the blood (HbA1c ≥6.5%), while controls were assigned as HbA1c≤5.6%. Peptides were identified using mass spectrometry, measured in the DDA mode, and processed using MaxQuant.66 against the decoy UniProt-human database. Finally, from the differential analysis for all tissues and comparisons, pancreatic islets and T2D-control samples were selected where the statistically significant data were defined by a *p* value < 0.05, resulting in 92 proteins.

### 2.4 Multi-Omics Data Integration

OmicsNet 2.0 [32] is a tool that allows the addition of one or multiple lists of genes, mRNA, proteins, *etc.*, facilitating the creation of various biological networks and the analysis of enriched metabolic pathways and gene ontology (GO). The data previously obtained for T2D were uploaded using the *H. sapiens* (human) filter and the gene's official symbol. Subsequently, STRING was selected for the protein-protein interaction network. It was filtered by tissue “pancreas;” a degree of 25.0 was applied to all nodes and adjusted for better 2D visualization. For the enrichment analysis using gene ontology, in the “Function Explorer” section, “all nodes” were performed, and the top ten were selected for each component of gene ontology and KEGG pathways. The same procedure was applied for each module, created by the infoMap algorithm, and the top five KEGG pathways were chosen. The hub genes were determined according to their degree, and the top ten were selected, representing the genes with the most interactions.

### 2.5 Virtual Screening

The top ten hub genes obtained were selected for the virtual screening analysis, and the PDB format files of proteins were downloaded from the Alphafold Database [33]. The results were filtered to include only those structures related to the *Homo sapiens* species, and only the reviewed structures were considered. Subsequently, a virtual screening was conducted using DrugRep [34], a parameter-free server for drug repurposing based on receptor interactions. This fully automated process included molecular 3D structure construction, binding pocket prediction, docking, and other steps to assess the interaction between proteins and an experimental drug library. The analysis was performed using the experimental drug library.

### 2.6 Molecular Docking Analysis

Twelve experimental drugs were chosen from the virtual screening to execute the molecular docking analysis with the ten hub genes. The SDF files of the experimental drugs were downloaded from the PubChem resource [35]. Then, through the CB-Dock2 [36] server, the protein-ligand blind docking between SDF files of experimental drugs and PDB format files of proteins previously downloaded from the Alphafold Database was performed. Lastly, the results were displayed using BIOVIA software [37] (Supplementary material).

## 3 RESULTS

### 3.1 Multi-omics Data Integration and Functional Enrichment Analysis

**Fig. (1):**
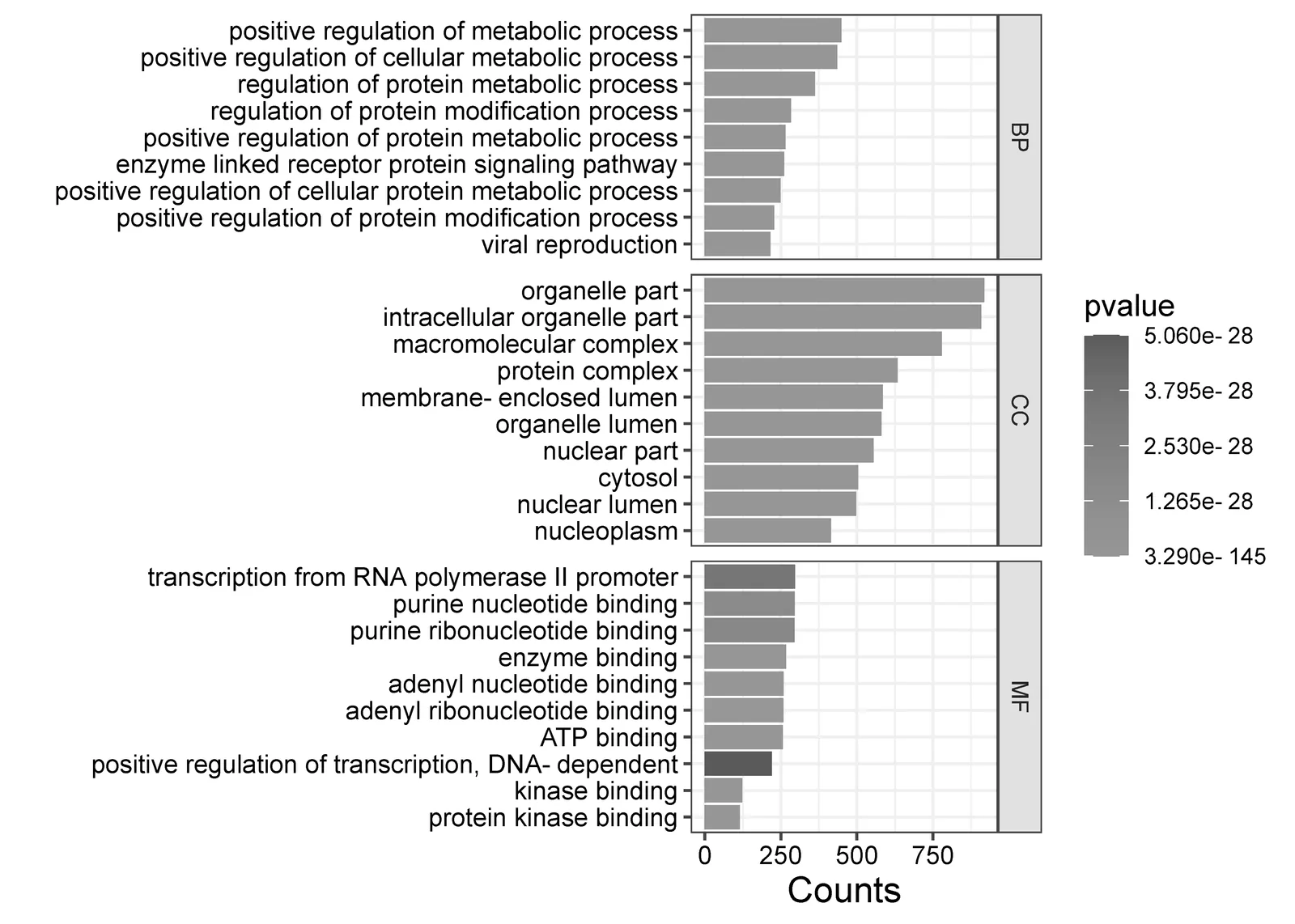
Gene ontology enrichment analysis of multi-omics data associated with T2D in pancreatic tissue. Biological Process, Cellular Component, and Molecular Function.

**Fig. (2):**
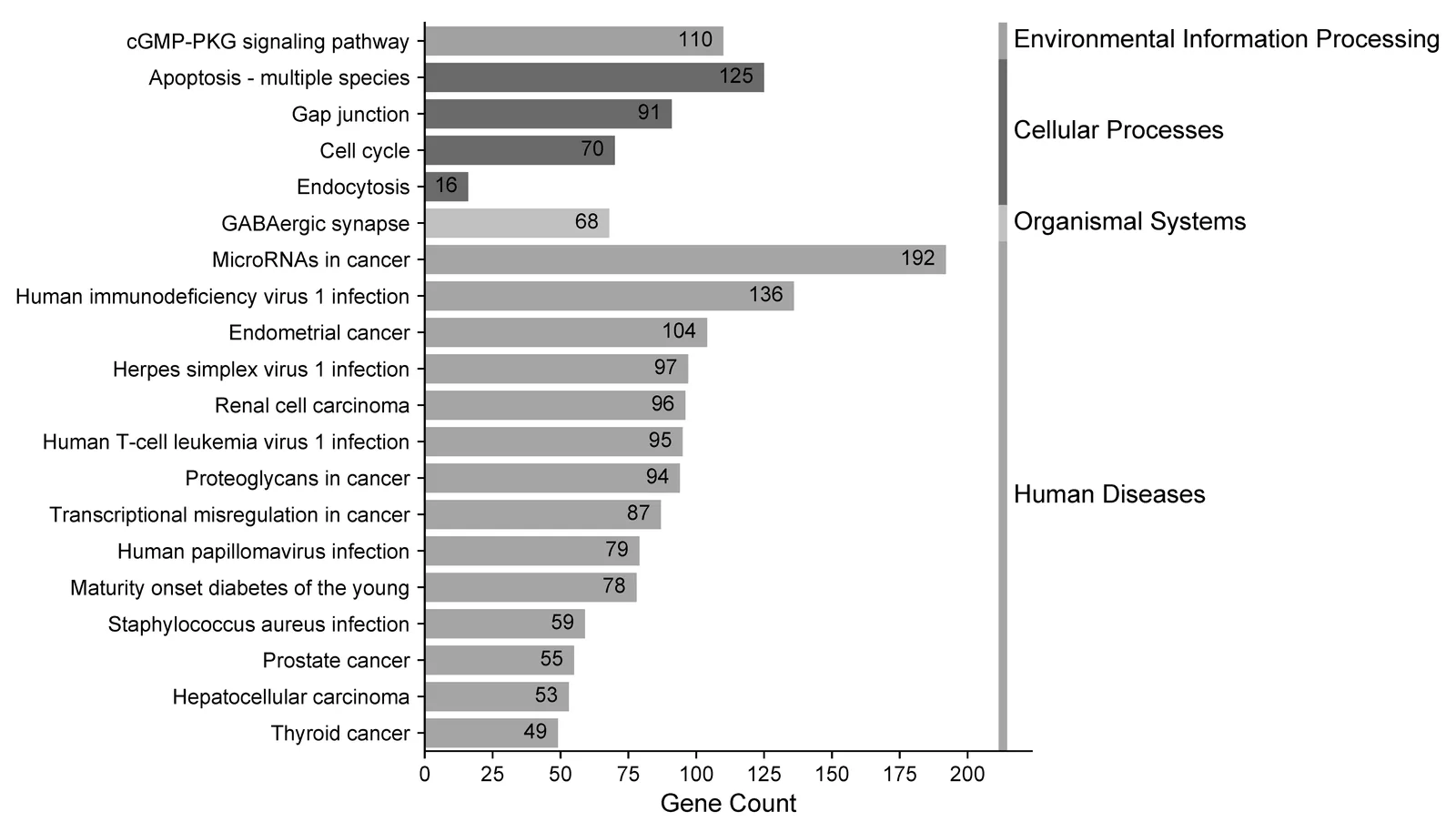
KEGG pathways enrichment analysis of multi-omics data associated with T2D in pancreatic tissue.

Using the omics data, 3260 genes, 6918 mRNA, and 92 proteins associated with T2D in pancreatic islets were found and integrated using OmicsNet 2.0. Then, we carried out the GO and KEGG pathway analyses according to the highest hits. Fig. (**1**). shows the main biological process implicated, underlining the positive regulation of the metabolic process, positive regulation of the cellular metabolic process, and protein metabolic process. As shown in Fig. (**1**), the cellular components involved were the organelle part, intracellular organelle part, and macromolecular complex. Also, molecular functions were primarily enriched in transcription by RNA polymerase II promoter, purine ribonucleotide binding, and purine nucleotide binding (Fig. **1**). Finally, KEGG analysis results indicated microRNAs in cancer, human immunodeficiency virus infection, and endometrial cancer (Fig. **2**).

### 3.2 Distribution by Modules of Multi-omics Data

After performing the enrichment analysis and KEGG pathways analysis on the total data, we distributed them by modules to reveal the primary mechanism involved. Fig. (**3**) shows the top five modules of KEGG pathways related to T2D in the pancreas, which are presented by color; green indicates gap junction and microRNAs in cancer, blue depicts basal primary transcription factors and DNA replication processes, pink represents modules mainly involved in renin secretion and cell cycle, while red and yellow indicate Ras/MAPK signaling pathways and oxidative phosphorylation/glutamatergic synapse, respectively.

### 3.3 Hub Genes and PPI Network

The protein-protein interaction network was performed using the STRING database in PPI OmicsNet 2.0, and the hub genes were obtained (Fig. **4**). Hub genes were determined as the top ten genes with the most interactions. The results of PPI indicated that Proto-oncogene non-receptor Tyrosine Kinase (SRC), Tumor Protein P53 (TP53), Serine/Threonine Kinase 1 (AKT1), Catenin Beta 1 (CTNNB1), Histone Acetyltransferase P300 (EP300), Mitogen-Activated Protein Kinase 1 (MAPK1), Mitogen-Activated Protein Kinase 3 (MAPK3), CREB Binding Protein (CREBBP), Epidermal Growth Factor (EGFR), and Heat Shock Protein 90 Alpha Family Class A Member 1 (HSP90AA1) had the most significant number of interactions.

### 3.4 Molecular Docking between Candidate Experimental Drugs and Hub Genes

The ten hub genes obtained from the PPI were selected to perform a virtual screening with experimental drugs for drug repurposing, ranked according to the docking scores, and exhibited as tables of drug information. Candidate experimental drugs were considered according to the lowest score and their possible action in most hub genes. Docking results between ten hub genes and experimental drugs presented by their ID Drug Bank are presented in Table **1**, where the lowest result indicated the best binding energy or a ΔG<0, expressed in kcal/mol. We found six good binding energies between five experimental drugs and four hub genes. The SD14 (DB02729) experimental drug bounded more strongly to SRC (ΔG=-12.1) and AKT1 (ΔG=-11). Moreover, voacamine (DB04877) showed good binding energy with SRC (G=-11.5) and the 5-[(2-methyl-5-{[3-(trifluoromethyl) phenyl] carbamoyl} phenyl) amino] pyridine-3-carboxamide (DB07970) experimental drug with this gene (ΔG=-11.1). On the other hand, the 1-[5-Methyl-2-(trifluoromethyl) furan-3-yl]-3-[5-[2-[[6-(1H-1,2,4-triazol-5-ylamino) pyrimidin-4-yl] amino] ethyl]-1,3-thiazol-2-yl] urea (DB07789) experimental drug indicated the best binding with CREBBP (ΔG=-11), and ZK-806711 (DB03373), the experimental drug, showed the lowest binding energy with HSP90AA1 (ΔG=-11.3). It is important to note that three drugs with a ΔG less than 11 targeted SRC protein. These complexes with the lowest binding energies underwent visualization to reveal the interaction between experimental drugs and the hub genes selected, as shown in Fig. (**5**).

## 4 DISCUSSION

This study aimed to explore and integrate multi-omics data related to T2D, focusing specifically on the pancreas. We identified various mechanisms involved, such as metabolic and transcriptional processes, through gene ontology, as well as pathways like Ras/MAPK signaling, gap junctions, and others, which were distributed across modules in KEGG pathways. Additionally, we selected ten genes with the highest interactions from the protein-protein interaction network to perform a virtual screening that revealed 12 candidate experimental drugs that docked most of the hub genes and presented good binding energies. Five experimental drugs and four hub genes were selected to execute the molecular docking, resulting in *SRC* with the lowest binding energies with three experimental drugs.

According to our results, the main biological processes involved were metabolic and cellular in T2D, where the pancreas was found to be mediated by these mechanisms, affecting early beta-cell dysfunction and death [38]. Insulin secretion is essential for controlling glycemia, allowing the entry of glucose as an energy source and, therefore, metabolic fuel homeostasis [39], which is potentially activated by a postprandial increase in glucose concentrations, along with the effects of amino acids, fatty acids, and hormones produced in α-cells and the gastrointestinal tract [40]. A study by Hu *et al*. [41] also found that the biological mechanisms involved were galactose metabolism, sugar metabolism, galactose metabolism, and glucose metabolism through an enrichment analysis in T2D and pancreatic tissue.

In this study, cellular components and molecular functions, such as intracellular and organelle parts, ATP binding, and enzyme binding, were found to be enriched in T2D and pancreatic beta-cells, where mitochondria play an essential role in insulin secretion like ATP production, Ca^2+^ signaling, ROS levels, *etc* [42]. It has been reported in mouse models that mitochondrial metabolism is impaired in diabetic islets, leading to a failure of glucose to elevate cytosolic ATP and drive GSIS [43]. On the other hand, glucose uptake is not rate-limiting for GSIS. It is also regulated by the glucokinase (GCK) enzyme, which acts as a glucose sensor [40] and determines the entry of glucose into the glycolytic pathway and insulin secretion where genetic variants cause partial or complete loss of enzyme activity, resulting in insulin insufficiency and potentially associated with an increased risk of T2D, according to a study conducted in Saudi Arabia [44]. So, in the future, it may help identify and classify individuals susceptible to T2D according to genetic variants.

**Fig. (3):**
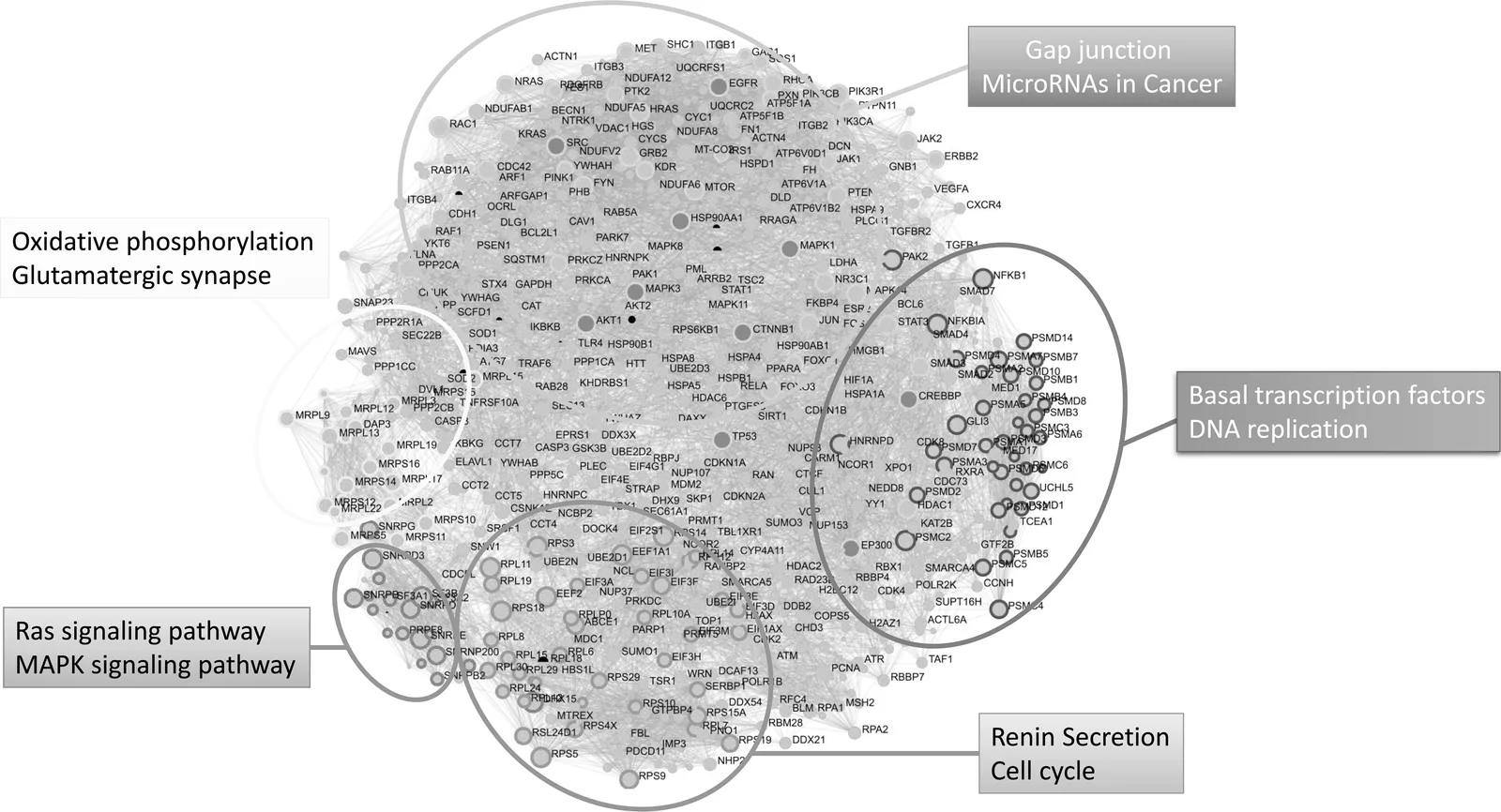
Modules of top five KEGG pathways related to T2D in pancreatic tissue.

**Fig. (4):**
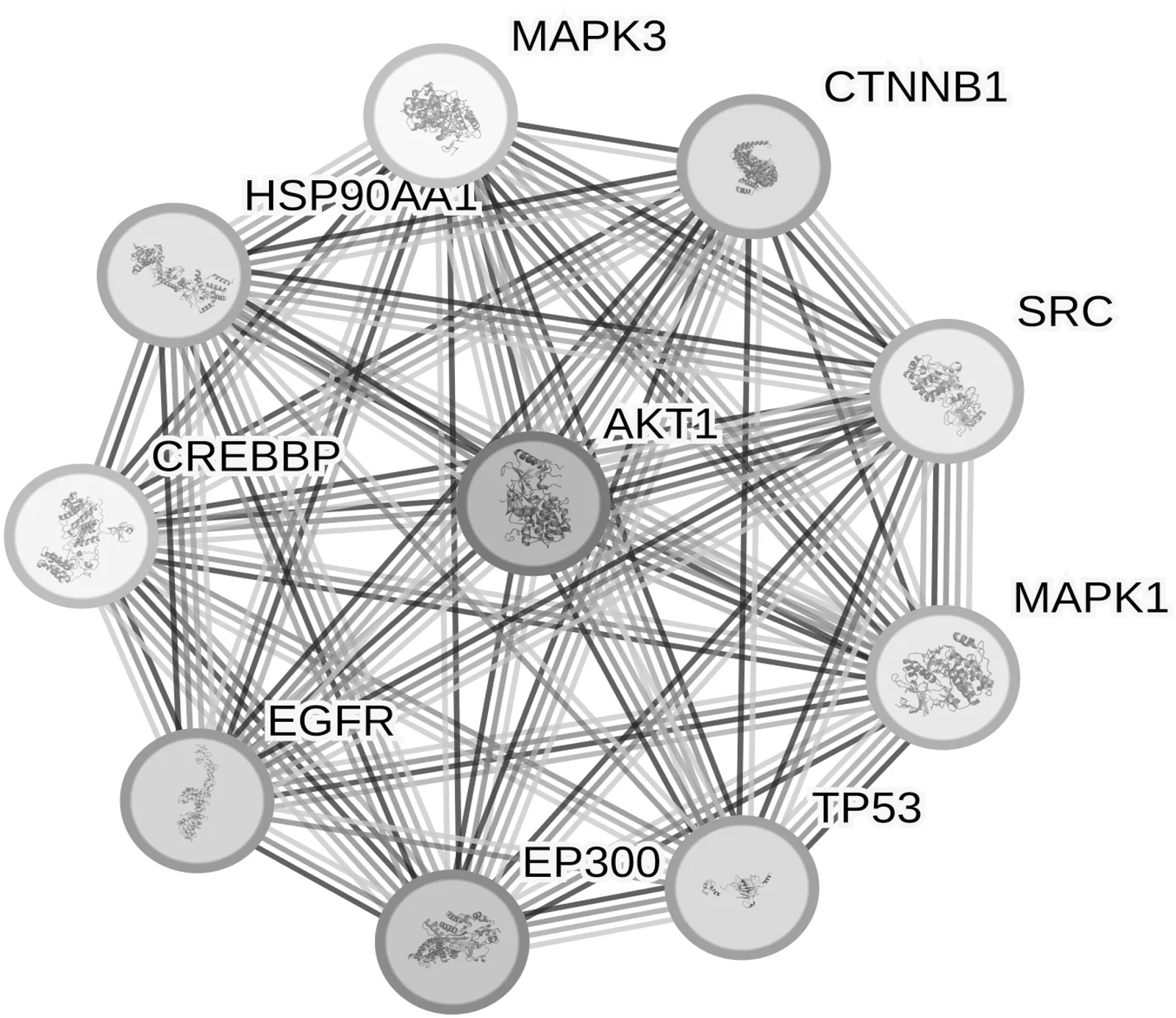
Protein-protein interaction network of hub genes associated with T2D in pancreatic tissue.

MicroRNAs (miRNAs) in cancer were found to be involved in KEGG pathways (Fig. **3**), and numerous studies have pointed to T2D as a known risk factor for pancreatic cancer [45], where the insulin released from beta-cells into the intrapancreatic portal circulation nourishes acinar and ductal cells adjacent to the islets, thus promoting their proliferation and increased risk of pancreatic cancer [46]. According to Yuan *et al.* [47], participants with recent-onset diabetes had an elevated risk for pancreatic cancer, which is particularly interesting to the oncology community, given that the increase in epidemiological data points to pancreatic cancer as a cause of diabetes due to unknown mechanisms [48].

The data distributed by modules also revealed transcription factors, DNA replication, gap junctions, and cell cycle, which are involved in the regulation of Ca^2+^ dynamics linked to the electrical activity of beta-cells that is important for insulin granule release and as secondary messenger can affect gene transcription [49] and several transcription factors, such as nuclear factor of activated T-cell (NAFT) that regulates genes involved in glucose sensing, beta-cell proliferation, and insulin release [50]. In contrast with the findings of a study by Miranda *et al.* [51], in human islets from donors with T2D, NAFT activation by glucose was diminished but rescued upon pharmacological stimulation of electrical activity, which regulated *GJD2* expression and increased gap junction permeability that coordinated Ca^2+^ regulation. Therefore, NAFT may affect target genes that mediate beta-cell proliferation and susceptibility to T2D.

According to the literature, Ras/MAPK signaling pathways are enriched, and insulin has a regulatory role in the insulin receptor (*INSR*) alternative splicing through the Ras/MAPK signaling pathways implicated in beta-cell mass and survival [52]. Insulin receptor isoform B (INSR-B) is predominantly expressed in pancreatic beta-cells, liver, muscle, adipose tissue, and kidney and also in metabolic signaling [53]. A study carried out on human and mouse islets found that an alternative splicing switch, which reduces INSR-B, is associated with increased insulin resistance and T2D coupled with high glucose and lipid levels, affecting subsequent insulin receptor signaling [54]. Therefore, INSR-B protects beta-cells from stress-induced cell death. It would be interesting to elucidate the mechanisms to increase these receptor levels in beta-cells to increase their protection.

The PPI network results showed SRC as the hub gene with the most interactions, which plays an important role in the overproduction of reactive oxygen species (ROS). Sato *et al*. [55] found that SRC downregulation reduced GSIS, decreased intracellular ATP content, and had a milder suppressive effect on Ca^2+^ elevation during K^+^ exposure in pancreatic islets of diabetic rats [56]. Therefore, it would be interesting to find a drug that modulates its activity. Besides, EGFR is a transmembrane receptor tyrosine kinase and is also one of the key targets for treating T2D, as reported in a study based on network pharmacology [57]. Some studies suggested that the lack of this receptor will lead to impaired pancreatic islet development in mice. If the EGFR signaling is weakened, beta-cell proliferation will be significantly inhibited [58].

On the other hand, CTNNB1 is considered necessary in beta-cell differentiation, where the deletion of this gene in Pdx1 pancreatic progenitors leads to decreased beta-cell mass and impaired glucose tolerance [59]. In contrast with an *in silico* study on differentially expressed genes in South Asian Populations (SAPs), CTNNB1 and RUNX2 hub genes were found to be unique for T2D pathogenesis [60]. However, at the same time, the increase in the mass of beta-cells plays a role in the compensatory phase during the early phase of T2D development [61], which is mainly mediated by AKT/GSK3-β/β-catenin and Tub signaling pathway [62]. Therefore, new therapeutic agents that inhibit this pathway may help prevent or retard the progression of T2D.

**Fig. (6):**
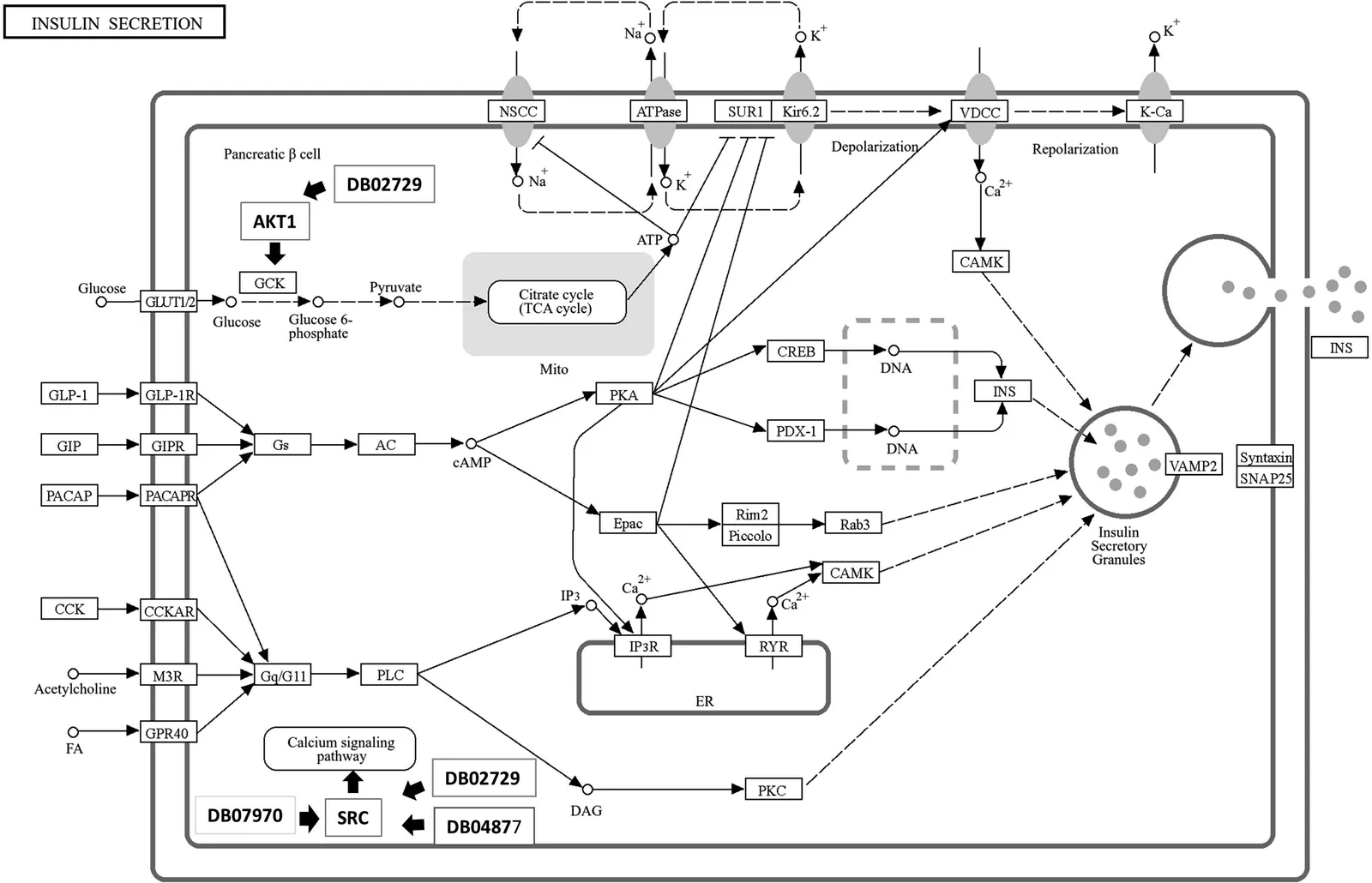
Possible mechanisms of action of DB02729, DB04877, and DB07970 on SRC and AKT1 for insulin secretion.

SRC was found to be the protein with the lowest binding energies with three experimental drugs. SRC-DB02729 complex demonstrated the best score, and according to the literature, this compound belongs to the class of organic compounds known as benzimidazoles. It has been studied as a possible antiretroviral in combination with other drugs and possibly capable of inhibiting a broad panel of drug-resistant variants [63]. We found that DB02729 binds to SRC and perhaps regulates it; previous studies have reported that this protein regulates insulin secretion from pancreatic cells by increasing cytosolic calcium. Therefore, DB02729 could regulate insulin secretion (Fig. **6**) [55, 64]. However, this compound demonstrated poor solubility and bioavailability, precluding further compound development [65].

The second complex with the best score was SRC-DB04877. Voacamine is a bisindole alkaloid isolated from *Voacanga* and *Peschiera species* in the Apocynaceae family. It consists of an iboga alkaloid skeleton, voacangine, directly connected to a 2-acyl indole unit, vobasine [66], which is responsible for its therapeutic properties, such as antimicrobial activity, neuroprotection, and the ability to counteract mutagenicity. It has also been widely studied as an anticancer agent in osteosarcoma [67]. This compound has previously been linked to diabetes by binding to PTP1B, thereby regulating the insulin signaling pathway. However, our results demonstrated that it may also influence insulin secretion by regulating SRC and DB02729 (Fig. **6**) [68].

DB07970 is an organic compound belonging to the benzenoid group. It is a poorly studied drug, and to our knowledge, no reports are linking it to T2D. However, according to our results, it is likely to regulate insulin secretion through SRC as DB04877 and DB02729 (Fig. **6**). Our results also showed that DB02729 can bind to AKT1. Although this drug has not previously been reported to be related to insulin secretion, one study suggests that reduced AKT1 expression induces a reduction of GCK, thereby regulating insulin secretion in pancreatic beta-cells (Fig. **6**) [69].

CREBBP, also known as CBP, is a therapeutic target for DB07789, a diazine drug. CBP and EP300 are transcriptional coactivators with histone acetyltransferase activity, which are involved in pancreatic beta and alpha cell proliferation and development [70, 71]. Wong *et al.* reported that mice lacking p300 or CBP in islets had reduced beta-cell area and insulin content [72]. Therefore, DB07789 could act on these genes to stimulate insulin secretion. Similarly, HSP90AA1 is a therapeutic target for DB03373, a type of benzenoid that may be able to activate HSP90AA1, which is involved in protein folding in response to glutathione deficiency as a key factor of unfolded or misfolded proteins in the endoplasmic reticulum (ER) [73] that contributes to oxidative stress, thus disrupting disulfide bond and aggregation of misfolded proteins and leading to pancreatic beta-cell dysfunction and the activation of apoptotic pathways in T2D [74, 75].

Fig. (**6**) illustrates the insulin secretion mechanism, where the experimental drugs DB02729, DB04877, and DB07970 may enhance SRC activity. This is in contrast to studies that report a downregulation of SRC activity in type 2 diabetes, suggesting that these experimental drugs could play a crucial role in modulating SRC activity and, consequently, influence the calcium signaling pathway involved in insulin secretion. It also shows the DB02729 experimental drug, which joined AKT1 and could increase its expression to induce an increase in GCK enzyme to enable glucose entry into the glycolytic pathway and regulation of insulin release.

The main limitation of this research is that it was based on an *in-silico* analysis. Therefore, further validation of the identified hub genes is still required based on laboratory experiments with pancreatic islet samples in humans with T2D, and the multi-omics data retrieved belongs to multiple ethnic groups, which may affect the variability of the results. At the same time, more research is required on the proposed experimental drugs. There is a limited number of proteomic studies on T2D in pancreatic islets compared to genomic and mRNA studies; nevertheless, we have chosen to include these studies to enhance the strength of our research and maintain the concept of multi-omics integration.

## CONCLUSION

The GO analysis and KEGG pathways analysis of the modules showed metabolic and cellular mechanisms like ATP binding and enzyme binding, miRNAs in cancer, transcription factors, gap junctions, and Ras/MAPK signaling pathways, which are related to T2D pathogenesis in pancreatic tissue, affecting processes, such as mitochondrial metabolism, GSIS, ATP production, Ca^2+^ signaling, gene transcription, insulin receptor, *etc.* We identified the hub genes SRC, TP53, AKT1, CTNNB1, EP300, MAPK1, MAPK3, CREBBP, EGFR, and HSP90AA1, which could provide useful information for future studies on significant pancreatic beta cell-associated genes that could have potential for targeted therapy of T2D. However, it is challenging to ascertain whether the discrepancy in the expression of these hub genes is a primary factor or an indirect consequence of the disease. SRC, AKT1, CREBBP, and HSP90AA1 were found to be the therapeutic targets for DB02729, DB04877, DB07970, DB07789, and DB03373. However, further studies are required to validate the hub genes and suggested experimental drugs.

## Figures and Tables

**Fig. (5):**
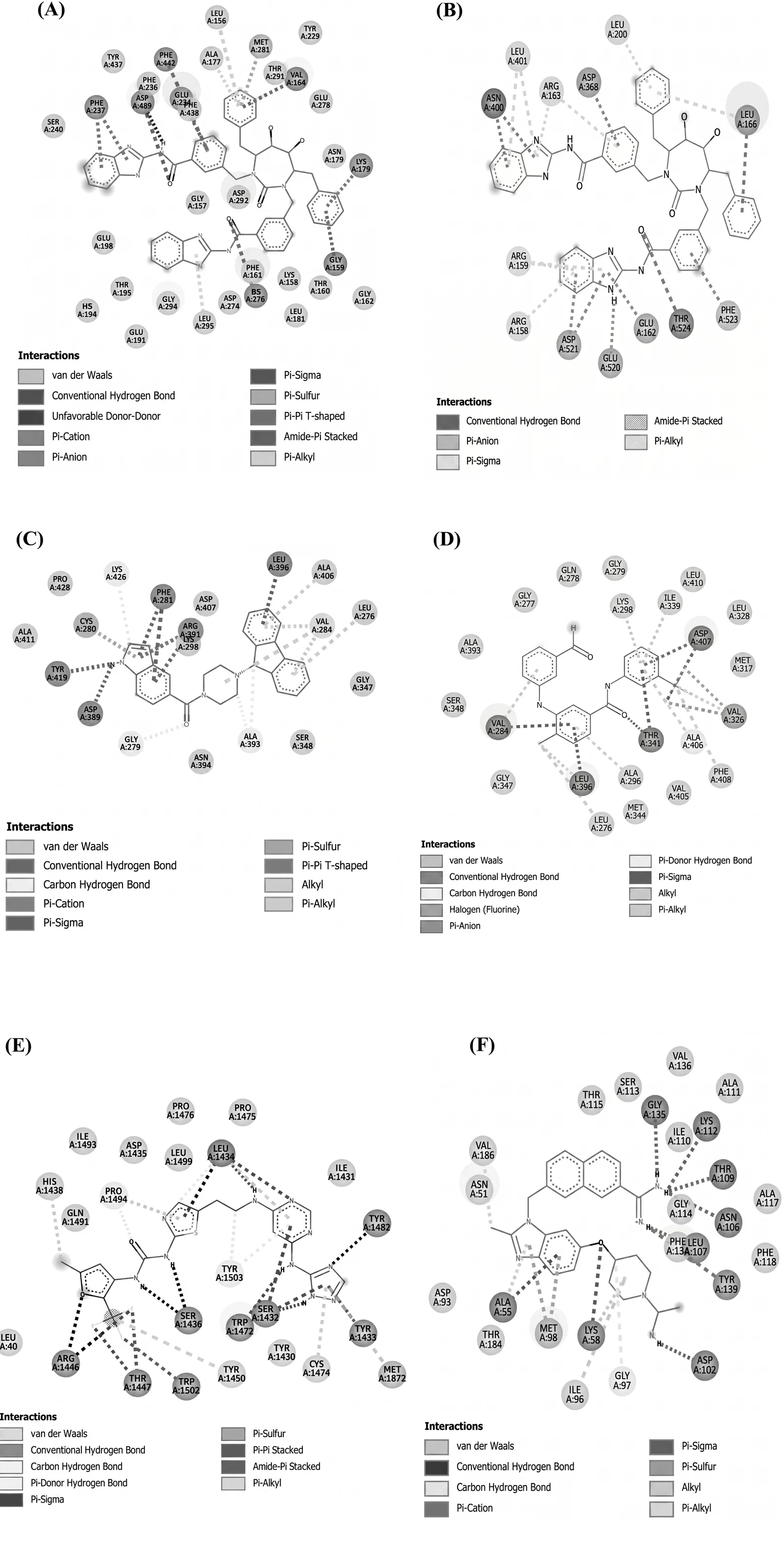
Interactions of the complexes with the best binding scores. **(A**) AKT1-DB02729, (**B**) SRC-DB02729, (**C**) SRC-DB04877, (**D**) SRC-DB07970, (**E**) CREBBP-DB07789, (**F**) HSP90AA1-DB03373.

**Table 1:** Scores from molecular docking between experimental drugs and top ten hub genes.

ID DRUG BANK	AKT1	CTNNB1	EP300	MAPK1	SRC	TP53	MAPK3	CREBBP	EGFR	HSP90AA1
DB02729	**-11**	-10.7	-10.5	-10.5	**-12.1**	-10.7	-9.9	-10.7	-9.3	-9.5
DB06925	-9.7	-10.2	-10	-9.5	-10.3	-9.7	-9.3	-12.6	-10.3	-10.3
DB04877	-8.3	-9.2	-9.1	-8.7	**-11.4**	-8.3	-9.4	-9.4	-9.3	-7.9
DB02226	-10.9	-10.7	-9.7	-9.1	-10.8	-9.3	-8.9	-10.6	-9.8	-10.1
DB04760	-10.3	-10	-8.8	-9.8	-10	-9	-9.7	-8.9	-10.1	-10.5
DB03373	-9.9	-8.9	-9.3	-9.2	-10.4	-9.4	-9.4	-9.3	-9.5	**-11.3**
DB04289	-9.8	-10	-8.9	-10.1	-11	-9.3	-9.1	-9.5	-10	-9.5
DB07789	-9.1	-9.2	-8	-8.3	-9.2	-8.7	-8.4	**-11**	-9.1	-10
DB04759	-9.5	-9.6	-8.7	-9.3	-9.8	-8.7	-9.2	-8.6	-9.4	-10.7
DB08730	-9.5	-9.7	-9	-9.4	-10.2	-9.3	-9.7	-9	-9.1	-9.7
DB13914	-9	-9.5	-9.1	-9.3	-10.2	-8.6	-8.9	-9	-9.7	-10.8
DB07970	-9.4	-9.7	-8.9	-9.5	**-11.1**	-8.9	-9.2	-10.5	-9.7	-10.2

Note: *Values are expressed in kcal/mol.

## Data Availability

The original contributions presented in the study are included in the article. Further inquiries can be directed to the corresponding author.

## LIST OF ABBREVIATIONS

GEO: Gene Expression Omnibus
CTD: Comparative Toxicogenomics Database
ER: Endoplasmic Reticulum
T2D: Type 2 Diabetes
